# *Bacillus spizizenii* DN and microbial consortia biostimulation followed by gamma irradiation for efficient textile wastewater treatment

**DOI:** 10.1007/s11356-022-24599-w

**Published:** 2022-12-11

**Authors:** Ola M. Gomaa, Shaimaa Abd El Mohsen Ibrahim, Nahla M. Mansour

**Affiliations:** 1 Radiation Microbiology Department, National Center for Radiation Research and Technology (NCRRT), Egyptian Atomic Energy Authority (EAEA), 3 Ahmad El Zomor St., Cairo, Egypt; 2 Gut Microbiota and Immunology Group, Chemistry of Natural and Microbial Products Department, Institute of Pharmaceutical Research Industries, National Research Centre, 33 El Bohouth St., Dokki, P.O. Box: 12622, Giza, Egypt

**Keywords:** Biostimulation, *Bacillus*, Textile wastewater, Gamma radiation, Microbial consortia

## Abstract

Textile wastewater still poses a huge environmental problem due to its high water consumption and high effluent release that is full of toxic chemicals. In the present study, different approaches were studied to layout an operating procedure for textile wastewater treatment in order to obtain treated wastewater that is safe for non-potable uses. Our approach depended on (1) co-substrate to biostimulate indigenous microbial textile wastewater community by adding Tryptone Soy Broth (TSB) and TSB supplemented with 1% glucose, (2) co-culture (bioaugmentation) with *Bacillus spizizenii* DN cells (previously isolated, identified and characterized as efficient decolorizing bacteria), and (3) co-metabolites using *Bacillus spizizenii* DN metabolites. The obtained results show that using *Bacillus spizizenii* DN cells resulted in 97.78% decolorization while adding *Bacillus spizizenii* DN metabolites resulted in 82.92% decolorization, both after 48 h incubation under microaerophilic conditions. The phyla identified for all treatments were Bacteroidota, Firmicutes, and Proteobacteria. The dynamic changes in the bacteria showed that both Clostridium and Acinetobacter disappeared for co-substrate, co-culture, and co-metabolite cultures. While Alkalibacterium and Stenotrophomonas appeared after adding *Bacillus spizizenii* DN cells, Flavobacterium increased for co-substrate and co-metabolic cultures while iron reducing bacteria appeared only for co-metabolic cultures. The use of 25 kGy gamma irradiation as a sterilization dose post bioremediation ensured safe use of treated wastewater. This was confirmed by cytotoxicity assay; the obtained IC_50_ tested on BJ fibroblasts obtained from skin showed that gamma irradiated treated wastewater are about 80.1% less toxic than non-irradiated treated wastewater. We conclude that (1) we can use combined bioaugmentation and biostimulation as initial steps for in situ bioremediation in collection tanks and that (2) the proposed protocol for bioremediation of industrial wastewater should be tailored based on the required application and level of safety needed for re-use.

**Graphical abstract:**

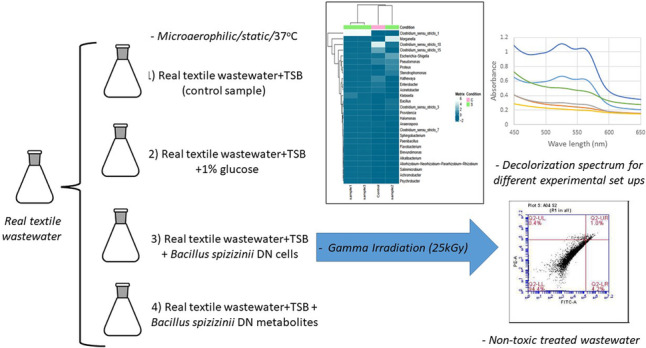

**Supplementary information:**

The online version contains supplementary material available at 10.1007/s11356-022-24599-w.

## Introduction

Textile industry is known to release huge amounts of water loaded with contaminants such as sizing agents, surfactants, fiber impurities, carboxylic acids, thickeners, complexing agents, salts, and dyes (EU report 2003). The common methods for treatment of textile wastewater can be physical, chemical, or biological methods; the latter is considered both cheap and efficient (Bilińska et al. 2016); in addition to that, it produces minimal sludge (Bidu et al. 2022). Biological methods usually take place through adsorption to cells such as bacteria, fungi, or microalgae. It can also take place via microbial degradation by cells or microbial enzymes. The composition of textile wastewater may contain from 10 to 200 mg/l dyes; in addition to other chemicals, this complex composition makes it highly toxic and, therefore, might affect the growth and activity of indigenous microorganisms (Jamee et al. 2019). There are different approaches to enhance the microbial degradation of textile wastewater: (1) biostimulation of indigenous microbiome by adding co-substrates. Nutrient scarcity limits the catabolic activity of indigenous microbial community, therefore, limiting the intrinsic bioremediation (Sarkar et al. 2016). Addition of nutrients can biologically stimulate the indigenous microbial community. Supplements can be carbon or nitrogen sources (Khan et al. 2013). (2) Bioaugmentation or what is also called co-cultures. The indigenous microflora can be stimulated by adding one or more microorganisms that support the growth of indigenous microflora. Microflora from rice husks was reported to induce dye decolorization and act as support material to enhance microbial community activity (Forss et al. 2017). (3) Co-metabolism which biostimulates the functionality of indigenous microbial community. The core microbiome in wastewater determines the efficiency of the bioremediation process (Carvalho et al. 2020), where the substrates fed influence the microbial growth, structure, dynamics, and bioremediation performance process through the presence of produced metabolites (Chen and Wu 2022). Those metabolites act as redox mediators which assist in the breakdown of dye molecules as well as other pollutants (Watanabe et al. 2009). It has been reported that redox mediators can be easily utilized by other microbes rather than their own (Rosenbaum et al. 2011). Riboflavin was reported to play a key role in dye degradation (Gomaa et al. 2019). Adding metabolites, such as cobalamin, to the medium can also shape the microbial community and direct its biodegradation performance (Chen and Wu 2022).

The *Bacillus* used in the following study was isolated and characterized in a previous study (Gomaa et al. 2021). The analysis of the whole genome of this bacterium revealed that it possesses genes for different xenobiotic degrading enzymes and metabolites such as riboflavin, cobalamin, and phenazine (Gomaa 2022). Bioaugmentation of *Bacillus spizizenii* DN or adding its metabolites to the textile wastewater is expected to enhance the indigenous microflora and enhance the metabolism of indigenous microflora, respectively.

To ensure efficient treatment for re-use, a sterilization step using ionizing radiation is proposed. The use of ionizing radiation provides a mode of breaking down organic compounds and disinfecting wastewater (Jiang et al. 2020). There has been many applications for using ionizing radiation for treatment of wastewater (Abdulrahman and Hung 2020) or sludge (Nakhla et al. 2022). Ionizing radiation can also be used as sterilization, it has been used successfully to remove bacteria from different samples. The sterilization dose was reported to be 25 kGy according to the International Standard Organization (Marsit et al. 2014). Therefore, the aim of the present work is to study the effect of adding (1) co-substrate, (2) co-culture, and (3) co-metabolism on decolorization and existing microbial flora. Gamma irradiation is used as a following step to sterilize the resultant-treated wastewater.

## Materials and methods

### Textile wastewater

Textile wastewater was obtained from a textile factory located at El Mahala Il Kobra. The effluent was obtained at the end of dyeing with red dyes. The effluent was collected in clean containers and sealed until the time of the experiment.

### Design of experiment and cultivation conditions

The textile dye was divided into four Erlenmeyer flasks containing 100 ml working volume, each flask represented a microcosm. Flask (1): the control (C) contained 40 ml textile dye and 20 ml Tryptone Soy Broth (TSB); flask (2): sample 1 (S1) contained 40 ml textile dye, 20 ml TSB, and 1% glucose; flask (3): sample 2 (S2) contained 40 ml textile dye, 20 ml TSB, and 4 ml 24 h grown *Bacillus* sp. cells; flask (4): sample 3 (S3) contained 40 ml textile dye, 20 ml TSB, and 4 ml filtered supernatant from an overnight culture of *Bacillus* sp. All flasks were incubated statically under microaerophilic conditions at 30 °C for 2 days. *Bacillus spizizenii* DN was previously isolated and used for nitrate containing textile wastewater (Gomaa et al. 2021). For co-cultures, a single colony of *Bacillus spizizenii* DN was used to inoculate Lauria Bertani media. The culture was incubated in shaker incubator (150 rpm) for 24 h at 30 °C. The culture was centrifuged at 6000 rpm for 15 min and 4 °C; the collected cells were used for co-cultures (bioaugmentation) while the culture filtrate was used as co-metabolites. All treated samples (1–4) were used to detect decolorization and analyze the microbiome as described below.

### UV–visible

A UV–visible scan was performed for each sample. The scan was performed from 200 to 800 nm using spectrophotometer (SPECORD 210 plus, analytic Jena). Decolorization was calculated according to the following equation:

$$\mathrm{Decolorization }(\mathrm{\%})=\frac{{A}_{i}-{A}_{f}}{{A}_{i}}$$

where *A*_*i*_ is the initial dye absorbance and *A*_*f*_ is the final dye absorbance.

### Fourier transform infrared spectroscopy (FTIR)

Fourier transform infrared spectroscopy (FTIR) of treated wastewater was studied after treatment with *Bacillus* sp. cells and that of *Bacillus* cells followed by gamma irradiation. FTIR was used to detect vibrational frequency changes. The obtained spectra represent the peaks for functional groups of the samples. Scanning was performed from 400 to 4000 nm using ATR-FTIR, BRUKER VERTEX 70 optics layout device at NCRRT. The analytical spectrum was then compared to the library to identify the functional groups.

### Microbiome analysis

#### DNA extraction

The microbial DNA was extracted using the PureLink™ Microbiome DNA Purification Kit (Invitrogen™, USA) following the manufacturer’s instructions. The extracted DNA was checked on 1% agarose gel electrophoresis followed by quantification using the 260/280 nm wavelength using Spectrophotometer 6715 (Jenway, UK).

### Library preparation and next-generation sequencing

DNA prepared samples was used as template in PCR reactions to amplify the V3–V4 hypervariable region of the 16S rRNA gene by using the set of primers (forward: 5′TCGTCGGCAGCGTCAGATGTGTATAAGAGACAGCCTACGGGNGGCWGC3′ and reverse: 5′GTCTCGTGGGCTCGGAGATGTGTATAAGAGACAGGACTA CHVGGGTATCTAATCC3′) in which bases indexes were incorporated to perform multiplexing. The PCR reactions were performed using PrimeSTAR R Max DNA Polymerase (Takara Kusatsu, Shiga, Japan). The PCR products were analyzed by gel electrophoresis for verification then purified with Ampure magnetic purification beads (Beckman Coulter, Atlanta, GA, USA). For the preparation of the libraries: the concentration of the PCR products was quantified by SYBR Gold Nucleic Acid Gel Stain, normalized, then pooled in preparation for bridge amplification. The final library was checked on a Bioanalyzer DNA 1000 chip for size verification which expected as ~ 630 bp. The sequencing of V3–V4 amplicons was carried out at the 57,357 hospital (Egypt) using MiSeq Illumina sequencer (Illumina, Inc., Madison, WI, USA) in 2X251 bp by using the Illumina MiSeq Reagent Kit v2 (500 cycles; Illumina) according to the manufacturer’s protocol.

### Bioinformatics analysis

The publically available tools such as FASTQC and FASTX were used for quality checking and quality-filtering followed by the package QIIME (Quantitative Insights Into Microbial Ecology) in addition to the package QWRAP (QIIME wrapper) to perform microbiome analysis.

Preprocessing and taxonomic identification were performed using dada2 workflow; filterAndTrim was used to filter and trim low-quality reads. Error rates were learned using nbases = 90,000. Filtered reads were merged and inferred using dada2 functions dada, mergePairs. Chimeras were removed. Generated sequence tables were imported to QIIME2. QIIME2 feature-classifier was to extract reads from silva-138–99 database with the parameters classifier-Naive-Bayes. The classification was performed using classify-sklearn. Diversity metrics were calculated using core-metrics-phylogenetic. QIIME2 was used to visualize the results in addition to R packages phyloseq and ggplot2 (Gao et al. 2022).

### Gamma radiation

Post-bioaugmentation with *Bacillus*, the decolorized sample was exposed to 25 kGy gamma irradiation for sterilization. The dose rate during the work was 1.33 kGy/h. Gamma irradiation was performed at the Canadian unit at the Co^60^ unit at the National Center for Radiation Research and Technology (NCRRT), Cairo, Egypt. Samples before and after gamma irradiation were analyzed using UV–visible and FTIR spectroscopy as described below.

### Toxicity

#### MTT assay

*Bacillus* sp. bioaugmentation and *Bacillus* sp. bioaugmentation and gamma irradiated samples were tested for their toxicity. BJ Cell Line cells were obtained from American Type Culture Collection; cells were cultured using DMEM (Invitrogen/Life Technologies) supplemented with 10% FBS (Hyclone), 10 µg/ml of insulin (Sigma), and 1% penicillin–streptomycin. All of the other chemicals and reagents were from Sigma or Invitrogen. Plate cells (cells density 1.2–1.8 × 10,000 cells/well) are in a volume of 100 μl complete growth medium + 100 μl of the tested compound per well in a 96-well plate for 24 h before the 3-(4, 5-dimethylthizaol-2-yl)-2, 5-diphenyl-2H-tetrazolium bromide (MTT) assay. Incubation is carried out for 48 h at 37 °C, read at 450 nm.

#### Apoptosis

We used FITC Annexin V Apoptosis Detection Kit I to determine the percentage of cells within a population that are actively undergoing apoptosis. Briefly, when cells undergo the apoptosis, the membrane phospholipid phosphatidylserine (PS) is translocated from the inner leaflet of the plasma membrane to the outer leaflet, and subsequently exposing PS to the external environment. Thus, Annexin V is a calcium-dependent phospholipid-binding protein which possess high affinity for the cells exposing PS. Additionally, propidium iodide (PI) is a standard flow cytometric viability probe, which can be used not only to stain the cells but also to distinguish viable from non-viable cells. This can be exemplified by viable cells with intact membranes excluding PI, whereas the membranes of dead and damaged cells are permeable to PI. Procedure according to FITC Annexin V Apoptosis Detection Kit I. Camptothecin stock solution (Sigma-Aldrich Cat. No. C-9911): 1 mM in DMSO and Jurkat T cells (ATCC TIB-152) are prepared prior to the experiment. For setting up compensation and quadrants, unstained cells were used as control, cells stained with FITC Annexin V (no PI) were positive control, and cells stained with PI (no FITC Annexin V) were used as negative control. Measurements were performed by BD Accuri C6 flow cytometer (USA).

## Results

### Decolorization

**Fig. 1:**
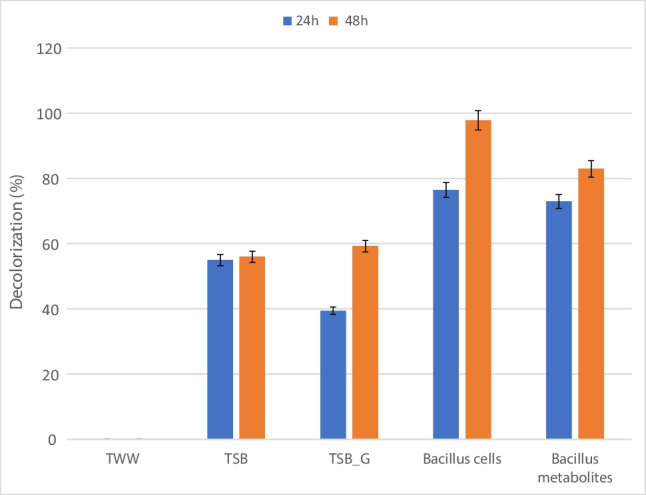
Decolorization of textile wastewater under different biostimulating conditions at 24 and 48 h

The decolorization of the four experimental setups showed different patterns. Figure 1 shows that adding TSB to the textile wastewater resulted in 54.95 and 55.95% decolorization after 24 and 48 h incubation, respectively. Adding glucose to textile wastewater resulted in an initial low decolorization after 24 h which was calculated to be 39.36%; this has increased to 59.26% after 48 h. The addition of 24 h grown *Bacillus spizizenii* DN to textile wastewater resulted in a visible color removal where the culture turned yellow within the first day of incubation; the decolorization reached 76.48 and 97.78% after 24 and 48 h incubation, respectively. On the other hand, *Bacillus spizizenii* DN metabolites resulted in 72.97 and 82.9% after 24 and 48 h incubation, respectively.

**Fig. 2:**
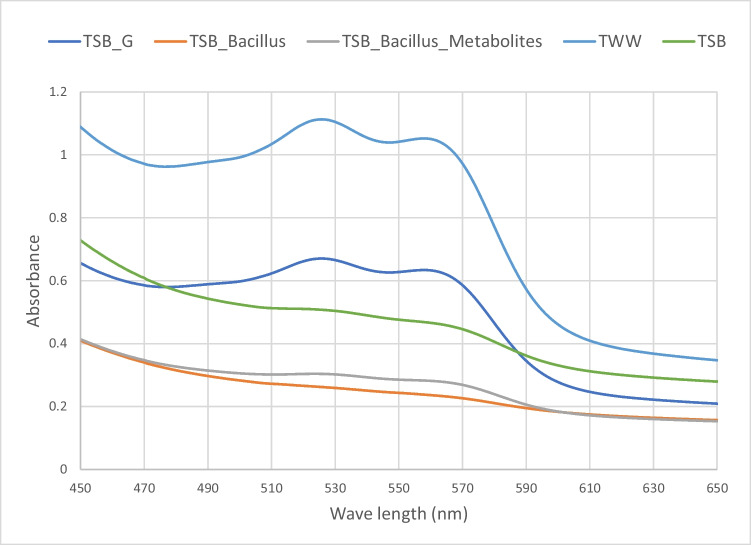
UV–visible spectrum of textile wastewater after treatment under different stimulating conditions

Figure 2 represents the changes in the UV–visible spectrum for dye before and after treatments. The spectrum shows the variation in dye removal which was adsorption in case of supplementing the textile wastewater with glucose where the assigned peaks for color were (525 and 560 nm) were still present, despite the lower absorbance, while the remaining treatments resulted in disappearance of those assigned peaks.

### Sequence analysis and microbial communities

**Fig. 3:**
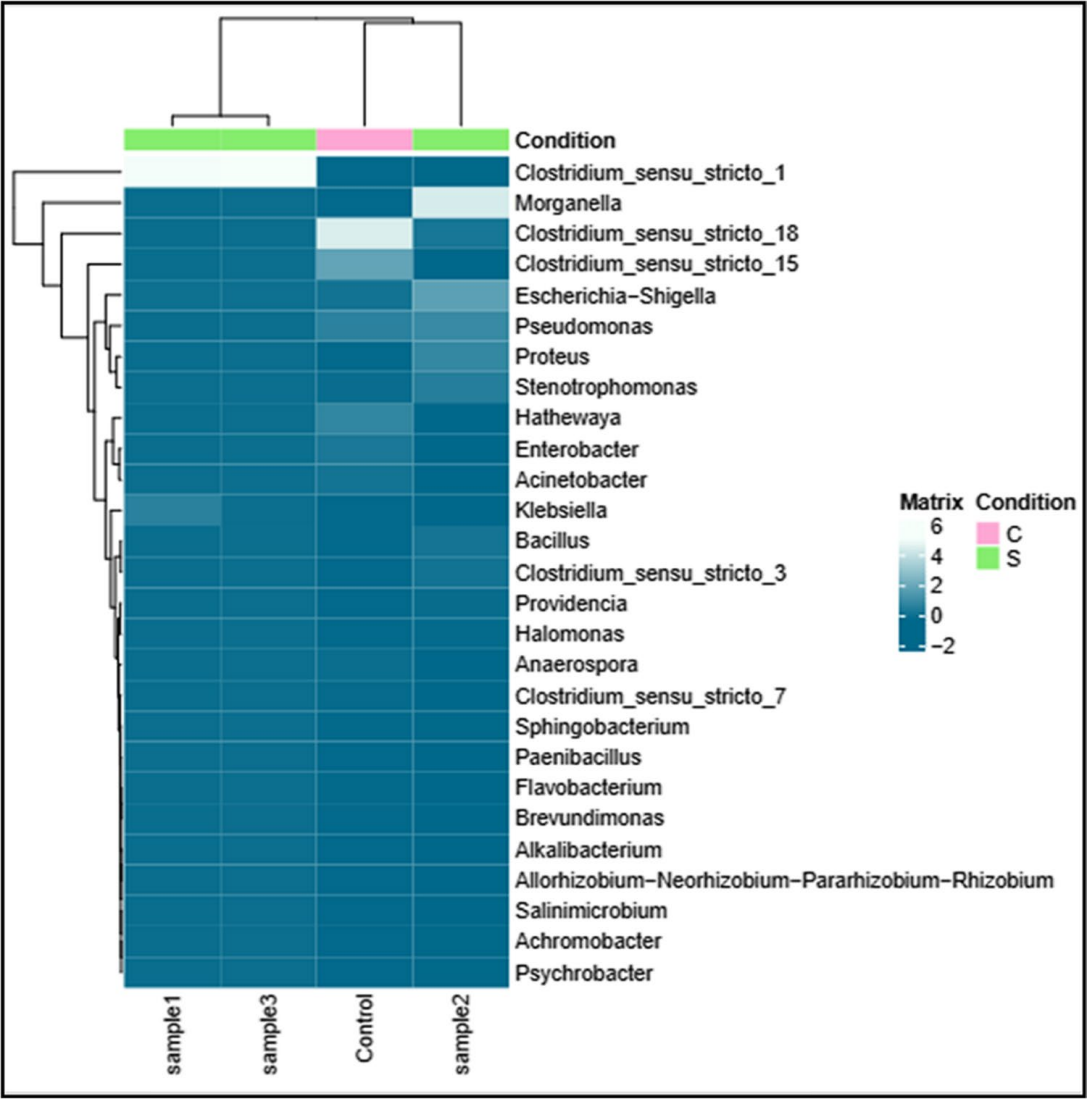
Heatmap of the microbial composition of the textile wastewater treatments (S1, S2, and S3) and control (C)

The hypervariable region V3-V4 of bacterial 16S gene from the control (C), S1, S2, and S3 was sequenced using MiSeq-Illumina system; the results showed 311,259, 365,119, 325,988, and 318,113 sequences per C, S1, S2, and S3 respectively. Figure 3 shows the relative abundance of major bacterial families detected for all the samples. The heatmap of the microbial composition among the three treatments (S1, S2, and S3) is compared to the control (C). The results show a display of available microorganisms for all the samples and their correlation to each other. Taxonomy details are displayed in Tables 1, 2, and 3.

**Table 1:** Phylum taxonomy

Phylum	Control	Sample 1	Sample2	Sample 3
Bacteroidota	240 (0.17%)	627 (0.24%)	303 (0.26%)	593 (0.24%)
Firmicutes	37,448 (26.87%)	106,296 (41.65%)	13,333 (11.64%)	105,973 (42.23%)
Proteobacteria	101,650 (72.95%)	148,292 (58.10%)	100,931 (88.10%)	144,386 (57.53%)
Unknown	0	0	1	0

**Table 2:** Species taxonomy

Genus/species	Control	Sample 1	Sample 2	Sample 3
*Flavobacterium lindanitolerans*	97	166	0	110
*Alkalibacterium iburiense*	0	0	55	0
*Iron-reducing bacterium*	0	133	0	11
*Clostridium* sp*.*	348	0	0	0
Uncultured bacterium	2	0	0	0
*Acinetobacter radioresistens*	1240	0	0	0
*Stenotrophomonas rhizophila*	0	0	650	0
Unknown	137,651	254,916	113,863	250,831

**Table 3:** Order taxonomy

Order	Control	Sample 1	Sample 2	Sample 3
*Flavobacteriales*	97	166	115	110
*Sphingobacteriales*	143	461	188	483
*Bacillales*	0	0	5680	186
*Lactobacillales*	0	0	55	0
*Paenibacillales*	0	883	0	0
*Clostridia*	1018	0	0	0
*Clostridiales*	35,589	105,413	7598	105,787
*Veillonellales-Selenomonadales*	793	0	0	0
*Caulobacterales*	57	0	0	0
*Rhizobiales*	0	0	0	4
*Burkholderiales*	0	0	185	0
*Enterobacterales*	94,965	146,894	83,242	143,919
*Oceanospirillales*	46	204	795	180
*Pseudomonadales*	5890	626	10,145	283
*Xanthomonadales*	692	544	6552	0
*Unknown*	48	24	13	0

Table 1 shows Proteobacteria as 72.95%, 58.10%, 88.10%, and 57.53% respectively followed by Firmicutes as 26.87%, 41.65%, 11.64%, and 42.23% respectively, while Bacteroidetes are presented in traces (0.17–0.26%). These phyla are commonly found in the microbial community of the textile wastewater.

### Gamma radiation for sterilizing the Bacillus-augmented-treated wastewater sample

**Fig. 4:**
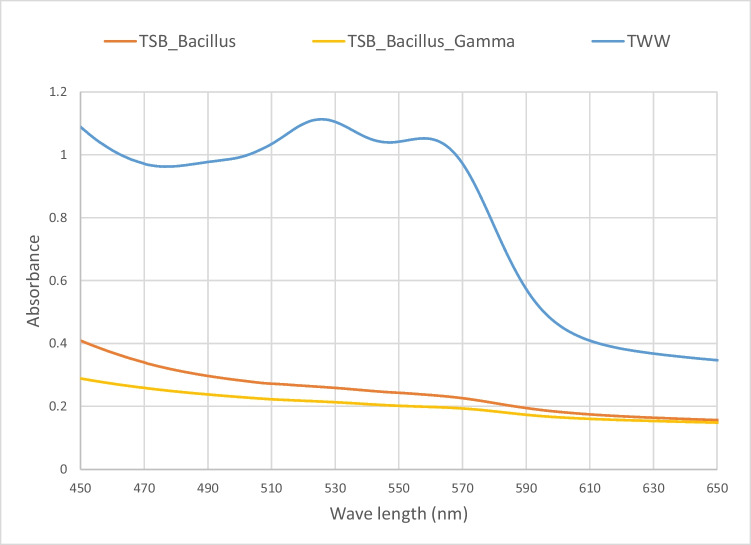
UV–Vis for *Bacillus* co-culture-treated samples before and after sterilization using gamma radiation

The aim of this experiment was to sterilize the resulting effluent before and after using gamma irradiation for sterilization. UV–Vis and FTIR spectra were obtained for both *Bacillus*-augmented samples and *Bacillus*-augmented and gamma-irradiated sample. The results show minimal changes between non-irradiated and gamma irradiated samples; this is an indication that gamma irradiation did not affect the content of the samples. On the other hand, there was no microbial growth on agar plates plated with gamma irradiated samples as compared to bacterial growth in non-irradiated samples (plate total count was 16 × 10^5^ cfu). The UV–visible spectroscopy shown in Fig. 4 shows a further decrease in the spectrum after irradiation; the decrease was about 4.26 and 4.56 for the peak at 525 nm and 5.55 and 5.2 for the peak at 560 nm for non-irradiated and gamma irradiated samples, respectively.

**Fig. 5:**
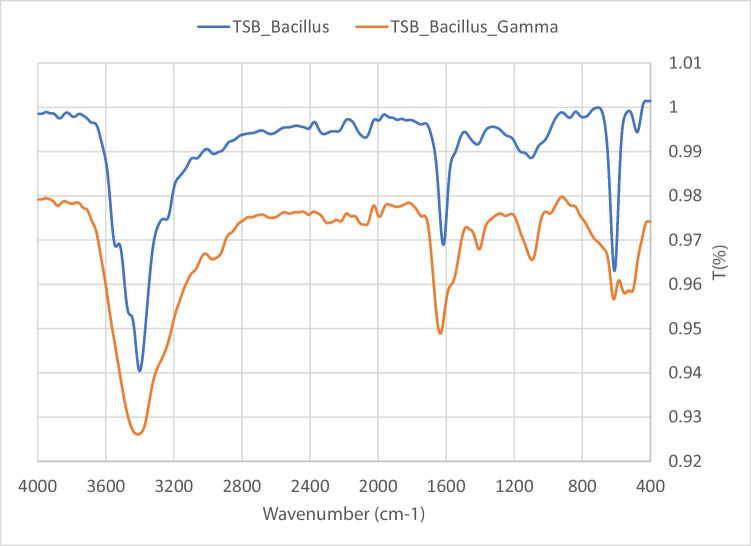
FTIR spectrum for *Bacillus* co-culture-treated samples before and after sterilization using gamma radiation

The obtained spectra shows that similar peaks for both gamma irradiated and non-irradiated sample peaks at 3400, 2962, 2266, and 2069 cm^−1^ are characteristic for OH, COOH, NH, and C≡C. Peaks shown at 1635 cm^−1^ represent C = O aldehyde; peaks at 1404 cm^−1^ represent COH bending. Peaks at 1093 cm^−1^ represent CO for ether or alcohol, and the fingerprint region shows peaks from 613 to 476 cm^−1^ representing halides. The results show that similar spectra suggest that there were no changes in the obtained treated wastewater for both *Bacillus*-augmented samples and *Bacillus*-augmented and gamma-irradiated sample (Fig. 5).

### Toxicity and quantitative measurement of the apoptotic cells using FITC Annexin V staining protocol

**Fig. 6:**
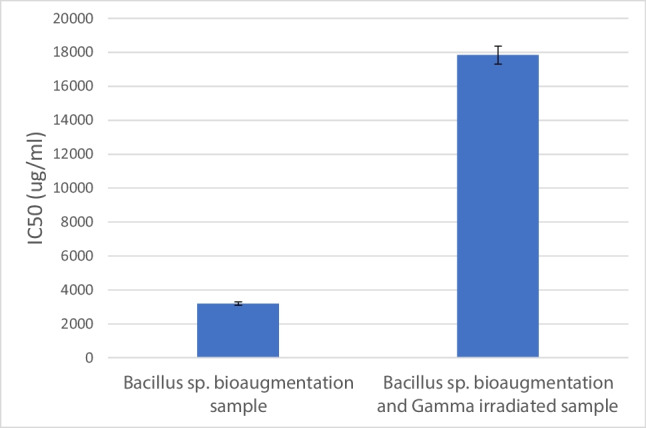
MTT assay for *Bacillus* sp. bioaugmentation sample vs. *Bacillus* sp. bioaugmentation sample followed by gamma radiation

**Fig. 7:**
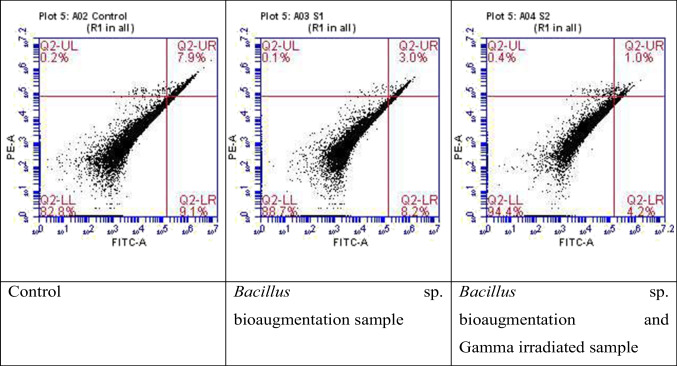
Apoptosis using PI and FITC fluorescent conjugate probes for samples before and after use of gamma irradiation

Testing the toxicity of both *Bacillus*-augmented samples and *Bacillus*-augmented and gamma-irradiated sample shows that the IC_50_ for non-irradiated sample was 3191.952 μg/ml while that for gamma-irradiated sample exceeded 17,835.12 μg/ml (Fig. 6). The apoptosis results showed double negative of 88.72 and 94.44% for *Bacillus*-augmented sample and gamma-irradiated sample, respectively as compared to 82.78% for control sample. Double-positive results showed 2.96 and 1.01% for *Bacillus*-augmented sample and gamma-irradiated sample, respectively, as compared to 7.86% for control sample (Fig. 7 and Table 4).

**Table 4:** Apoptosis data for samples before and after gamma irradiation

Sample	Control	*Bacillus* sp. bioaugmentation before γ irradiation	*Bacillus* sp. bioaugmentation after γ irradiation
UL (%) positive PE	0.25	0.09	0.37
UR (%) double positive	7.86	2.96	1.01
LL (%) double negative	82.78	88.72	94.44
LR (%) positive FITC	9.12	8.23	4.18

## Discussion

The microbiome in an environment is influenced by different factors such as the conditions of the sample, bioavailability of pollutants present, and indigenous microbes. This will determine their catabolic activity if it would be transformation, absorption, or mineralization (Haque et al. 2022). In the present study, the results obtained showed an increase in decolorization upon the addition of TSB media and glucose; despite the different mechanisms, TSB media components resulted in degradation as seen from lack of dye peaks. Sarkar et al. (2016) showed high microbial catabolic activity in the presence of high nitrogen source. On the other hand, addition of glucose resulted in adsorption as can be seen from the presence of dye peaks. This can be due to the catabolic repression effect of glucose which makes it difficult to utilize any other compound by inducer exclusion (Bren et al. 2016). On the other hand, the addition of *Bacillus* sp. as co-culture resulted in the highest dye degradation. Rahimi et al. (2020) states that both *Bacillus* sp. and wastewater microbial community enhanced the degradation process. According to other studies such as Kim et al. (2014) and Emmanuel et al. (2019), it was assumed that bioaugmentation to microbial consortia promote degradation, probably by linking more than one degradative and metabolic pathway (Santhanarajan et al. 2021). The third treatment approach was the use of metabolites produced by *Bacillus spizizenii* DN as co-metabolites to enhance biodegradation. The obtained results showed that adding the metabolites resulted in close degradation pattern but less than that obtained by adding *Bacillus spizizenii* DN cells to the wastewater. Adding metabolites to the wastewater affected the diversity of the microbiome and therefore the catabolic activity of the present microorganisms (Rosenbaum et al. 2011), and this has been reflected in the decolorization results obtained. It is very plausible that the decolorization result could be enhanced if the key metabolite involved in this process was identified. This is the focus of our upcoming work.

In addition to the complexity of textile effluents and their recalcitrant components, some factories combine domestic wastewater to textile wastewater prior to their release to public sewer systems. This is probably why our samples show unexpected bacteria that are commonly found in domestic wastewater such as gastrointestinal tract bacteria. Bacteriodetes are gram-negative anaerobes that inhabit the grastrointestinal tract, they digest carbohydrates through a series of metabolic pathways, and this fermentation of dietary material results in the formation of short chain fatty acids (Kirby et al. 2019). Firmicutes, also found in our sample, are gram-positive bacteria responsible for breakdown of dietary material and production of vitamins and short chain fatty acids such as butyrate (Ducret et al. 2021). Proteobacteria are gram-negative bacteria; they are found in different niche and are reported for their diverse application in bioremediation such as heavy metal tolerance and removal (Nyoyoko et al. 2022), utilization of wastewater for hydrogen, and electricity production; their metabolites produced during fermentation can act as redox mediators or help as quorum sensing molecules to enhance biofilm formation. Proteobacteria are recognized to have a significant role in the degradation of organic toxins such as nitrogenous, phosphorus, and aromatic compounds. The present bacteria do not only decolorize textile wastewater efficiently, but they also perform this decolorization under the extreme conditions of textile wastewater. Factors like high salinity, high alkalinity, and high temperature interfere with the decolorization process; therefore, a microbial consortium consisting of different halotolerant, alklitolerant, and thermotolerant bacteria is important for a successful treatment (Iqbal et al. 2022). Firmicutes abundance was reported under high salinity and high alkalinity; this indicated tolerance to extreme conditions in a batch reactor used for dye decolorization (Cao et al. 2019). Tizazu et al. (2022) reported Bacteroidota and Proteobacteria as abundant phyla in textile wastewater treatment plants that grew and decolorized textile dyes under extreme conditions of high salt, high temperature, and high alkalinity. The abundance of the same phyla in the present study suggests that the decolorizing activity of the indigenous microbial consortia is the best option for dye degradation under the extreme conditions of real textile wastewater.

To complete the treatment and remove any residues or bacteria, 25 kGy was used, *Bacillus spizizenii* DN-bioaugmented-treated wastewater was exposed to gamma irradiation. FTIR spectra show that samples before and after gamma irradiation were close. This indicates that gamma irradiation role was to ensure microbe-free-treated wastewater; this is expected since the sterilization dose used is 25 kGy (Marsit et al. 2014). This is another indication that the treatment protocol or operating procedure can dictate what type of application the treated wastewater can be used for.

This result is confirmed by testing the toxicity of the samples on dermal fibroblasts. The obtained data in the present work show a decrease in toxicity that reached its lowest when gamma irradiation was applied after biological wastewater treatment. A recent scientometric study reviewed textile toxicity trend; the study concluded that decolorization does not guarantee lack of toxicity (Vasconcelos et al. 2022). Studying the toxicity after textile dye wastewater is considered an important parameter due to its potential effect on plants, animals, and eventually human beings (Dhaouefi et al. 2022). Choosing the wastewater treatment approach depends on the required application.

## Conclusion

The complexity and toxicity of compounds present in textile wastewater calls for efficient treatment prior to their release. Understanding the textile wastewater microbial community under different conditions is crucial for in situ bioremediation. The present work highlights the dynamic changes in the microbial consortium after co-substrate, co-culture, and co-metabolite addition. This method can direct the catabolic activity of the indigenous microorganisms that in turn reaches maximal wastewater treatment. *Bacillus spizizenii* DN and its metabolites showed influence on the indigenous textile wastewater consortia. Gamma radiation can be used for effective sterilization if the treated wastewater is used in any activity that involves human skin exposure.

## Supplementary information

Below is the link to the electronic supplementary material.

Supplementary file1 (DOCX 180 KB)

## Data Availability

The obtained data will be available upon request.
