# Supplementary material for: Bacillus spizizenii DN and microbial consortia biostimulation followed by gamma irradiation for efficient textile wastewater treatment
Source: Environ Sci Pollut Res Int. 2022 Dec 11;30(12):33907–16. doi: 10.1007/s11356-022-24599-w (PMC10017596; doi:10.1007/s11356-022-24599-w)

**Fig. (1): Alpha diversity**


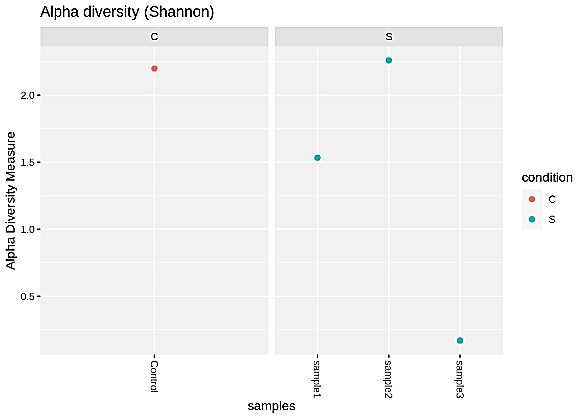

**Fig. (2): Beta diverse**

**Fig 3:** **Stacked bar charts of Relative abundance (%) of the genera level in the wastewater treatment (samples 1, 2, 3) compared to the control (C).**


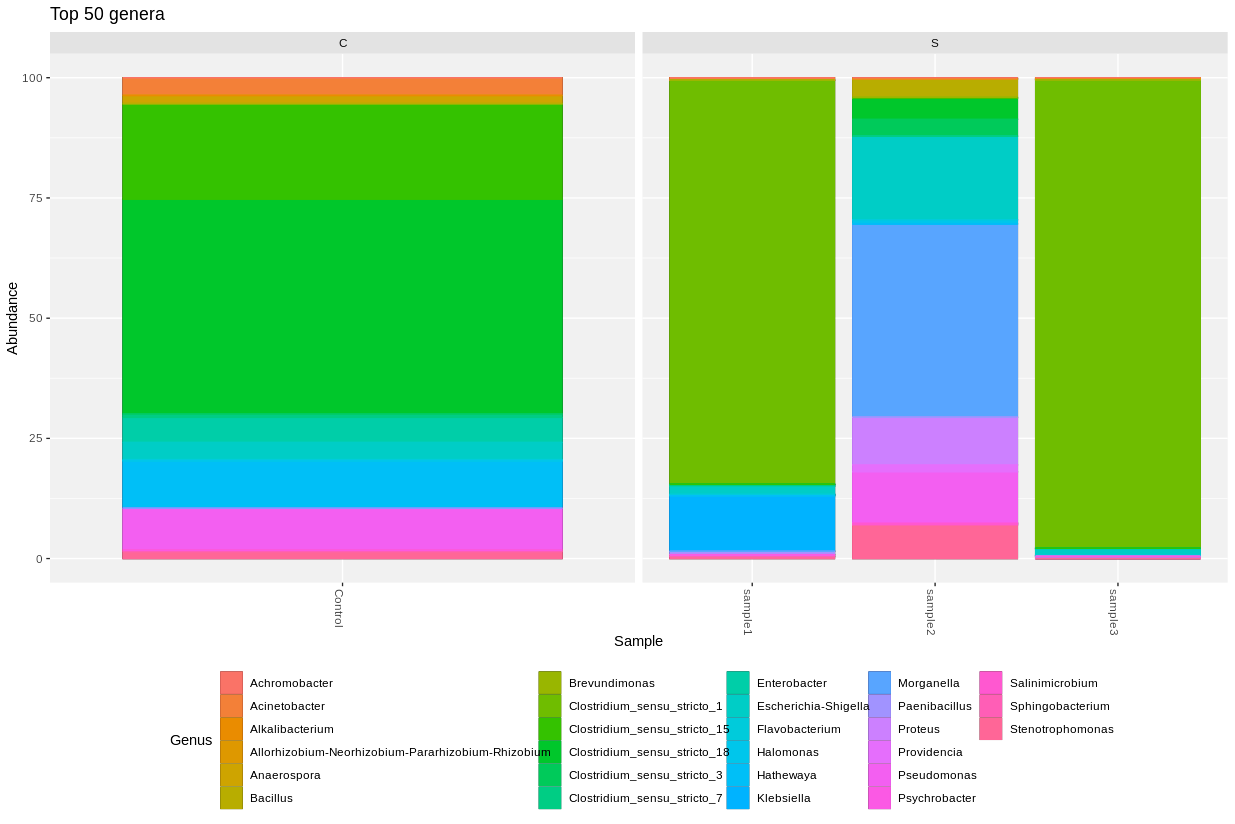

Supplement: Supplementary file 1 — Supplementary file1 (DOCX 180 KB) [file 11356_2022_24599_MOESM1_ESM.docx]
